# Research advances in red ginseng polysaccharides: preparation, structural characteristics and immunomodulatory effect

**DOI:** 10.3389/fphar.2026.1929729

**Published:** 2026-09-16

**Authors:** Xinpeng Yang, Pan Hu, Wei Han, Lili Dong, Hongyan Zhang, Jiahui Wang, Huijun Chen

**Affiliations:** The Second Affiliated Hospital of Heilongjiang University of Chinese Medicine, Harbin, China

**Keywords:** immunomodulatory effects, *Panax ginseng* C.A. Meyer, polysaccharide, red ginseng, structural characterization

## Abstract

Red ginseng is a processed product of ginseng through steaming. Its polysaccharides differ in structure and biological activity from those of ginseng. Red ginseng polysaccharides are mostly acidic polysaccharides and have various biological activities. It is worth noting that its immunomodulatory effect has become an important area of research. Pharmacological studies have shown that polysaccharides from red ginseng can enhance immunity through various pathways, such as affecting immune cell activity, regulating cytokine levels, promoting immune organ development, and regulating gut microbiota. In addition, red ginseng polysaccharide has other pharmacological effects, such as anti-diabetic effect, anti-Alzheimer’s disease effect, health benefits for the skin, anticancer effect, and cardioprotective effect. This article reviews the extraction, purification, structural characterization, and biological activity of polysaccharides from red ginseng to elucidate their pharmacological effects and potential molecular mechanisms. In addition, this review also discusses the current research status of red ginseng polysaccharides, pointed out the existing gaps, and proposed innovative perspectives for better development of red ginseng polysaccharides.

## 1 Introduction

Ginseng, the root and rhizome of *Panax ginseng* C.A. Meyer., is a perennial plant in the Araliaceae family. It has been used as a precious traditional Chinese medicine for thousands of years (Liu et al., 2024; Zhou et al., 2024). In China, it is listed as a second-class protected plant in the ‘List of National Key Protected Wild Plants in China’. Ginseng mainly grow in China, Korea, and Japan (Ito and Ito, 2024). It is widely used in the field of medicine due to its ability to enhance immunity, as well as its outstanding effects on the nervous system, endocrine system, and blood regulation (Niu et al., 2025; Wang and Xin, 2025; Wu et al., 2025). Ginseng is included in the Chinese Pharmacopoeia (2025 edition) (Chinese Pharmacopoeia Commission, 2025). In addition, it is also included in the European Pharmacopoeia, United States Pharmacopeia, Korean Pharmacopoeia, and Japanese Pharmacopoeia (Li et al., 2022). There are many processed products of ginseng, among which steaming fresh ginseng and then drying it to make processed products is called “red ginseng” (Zhu et al., 2026; Jeong et al., 2020) (Figure 1). Because ginseng undergoes these steps and turns red, it is named this way. This processing method improves the shelf life of ginseng and facilitates long-term storage of ginseng. At the same time, it also led to changes in the chemical composition and pharmacological activity of ginseng (Truong and Jeong 2022).

**FIGURE 1:**
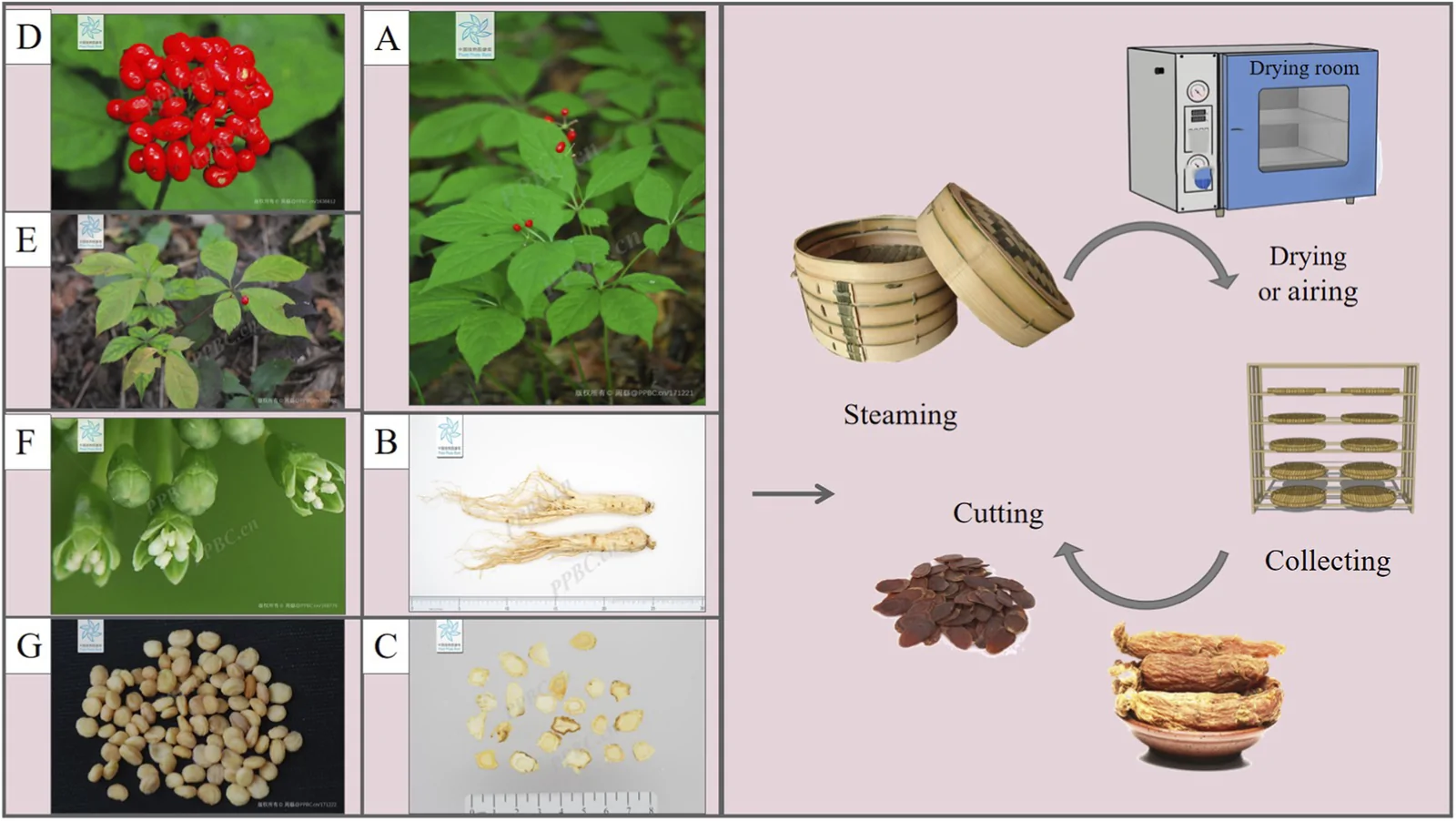
Plant morphology of *Panax ginseng* C.A. Meyer. **(A)** The aboveground part of the plant. **(B)** Root and rhizome. **(C)** Chinese herbal pieces. **(D)** Fruit. **(E)** Leaf. **(F)** Flower. **(G)** Seed. (Pictures are from public sources and the Internet).

Compared with ginseng, red ginseng has unique effects. In addition to having similar nourishing and tonifying effects as ginseng, it also shows beneficial effects in enhancing immunity, relieving fatigue, improving memory, and improving symptoms and functions in menopausal women (Ye et al., 2023). Moreover, the medicinal value of red ginseng is not only reflected in its individual use, but also in its synergistic effect when combined with other traditional Chinese medicines. Red ginseng is contained in traditional Chinese patent medicines such as Xiaokeling tablet, Tangniaole capsule and Shenfu injection solution (Qi et al., 2010; Huang et al., 2025). In addition, red ginseng is also a characteristic ingredient that enhances dietary nutrition and flavor in daily life. Since the implementation of the Korean Health Functional Foods Act in 2004, the efficacy of red ginseng as a healthy functional food has been certified (So et al., 2018). The China National Center for Food Safety Risk Assessment indicates that ginseng (artificially cultivated for 5 years or less) is a new resource food, and red ginseng processed from ginseng that meets this requirement can be used as a food ingredient. Plant based beverages, concentrated drinks, gummies, milk powder, and other products containing red ginseng have emerged one after another (Kim and Lee, 2024).

Red ginseng contains various chemical components, such as saponins, volatile oils, polysaccharides, amino acids, trace elements, etc (Cho et al., 2023; Wang et al., 2024). Polysaccharides, as natural bioactive substances, are widely present in the roots, stems, leaves, fruits, and other parts of red ginseng (Lee et al., 2015). Red ginseng polysaccharides play an important role in natural medicine research and functional food development. It has shown certain effects in immunomodulatory, anti-diabetic, anti-Alzheimer’s disease, health benefits for the skin, anticancer, and cardioprotective effect (Kim et al., 2019; Lee et al., 2020; Shin et al., 2021; Zhai et al., 2022). The pharmacological effects of red ginseng polysaccharides mainly depend on their chemical structure. The structure of red ginseng polysaccharides is complex, and different processing techniques and extraction methods can lead to differences in their chemical structure and biological activity. Due to its diverse pharmacological activities and low adverse reactions, red ginseng polysaccharides have become a hot topic in the development and application research of natural medicines.

For a long time in research, red ginseng has been mainly composed of ginsenosides as its active ingredient (Wang et al., 2024). In recent years, research on polysaccharides has gradually increased, but there is a lack of systematic sorting and summary. This article is based on the current research results of red ginseng polysaccharides, combined with production practice and research hotspots, to review the extraction and purification, structural characteristics, pharmacological effects, and structure-activity relationship of red ginseng polysaccharides, to provide reference for further research and industrial application of red ginseng polysaccharides.

## 2 Methods

The literature data was obtained from mainstream databases such as Web of Science, Scopus, ScienceDirect, SpringerLink, and PubMed in 2026. The search terms include “Red ginseng”, “Korean red ginseng”, “Ginseng Radix et Rhizome Rubra”, “Steamed ginseng”, “*Panax ginseng*” “*Panax ginseng C.A.* Meyer.”, “Polysaccharide”, “Acidic polysaccharide”, “Extraction”, “Isolation”, “Structure”, “Activity”, “Immunomodulatory effect”. Using a combination of topic words and free words, construct a search equation using Boolean operators (AND/OR/NOT). The language for literature search is English, and references are selected based on their relevance. Exclude duplicate studies and irrelevant references and review the abstracts of the remaining articles to ensure they meet the inclusion criteria of the review. At the same time, manual retrieval of relevant literature reviews is supplemented to obtain as comprehensive a list of relevant literature as possible (Figure 2).

**FIGURE 2:**
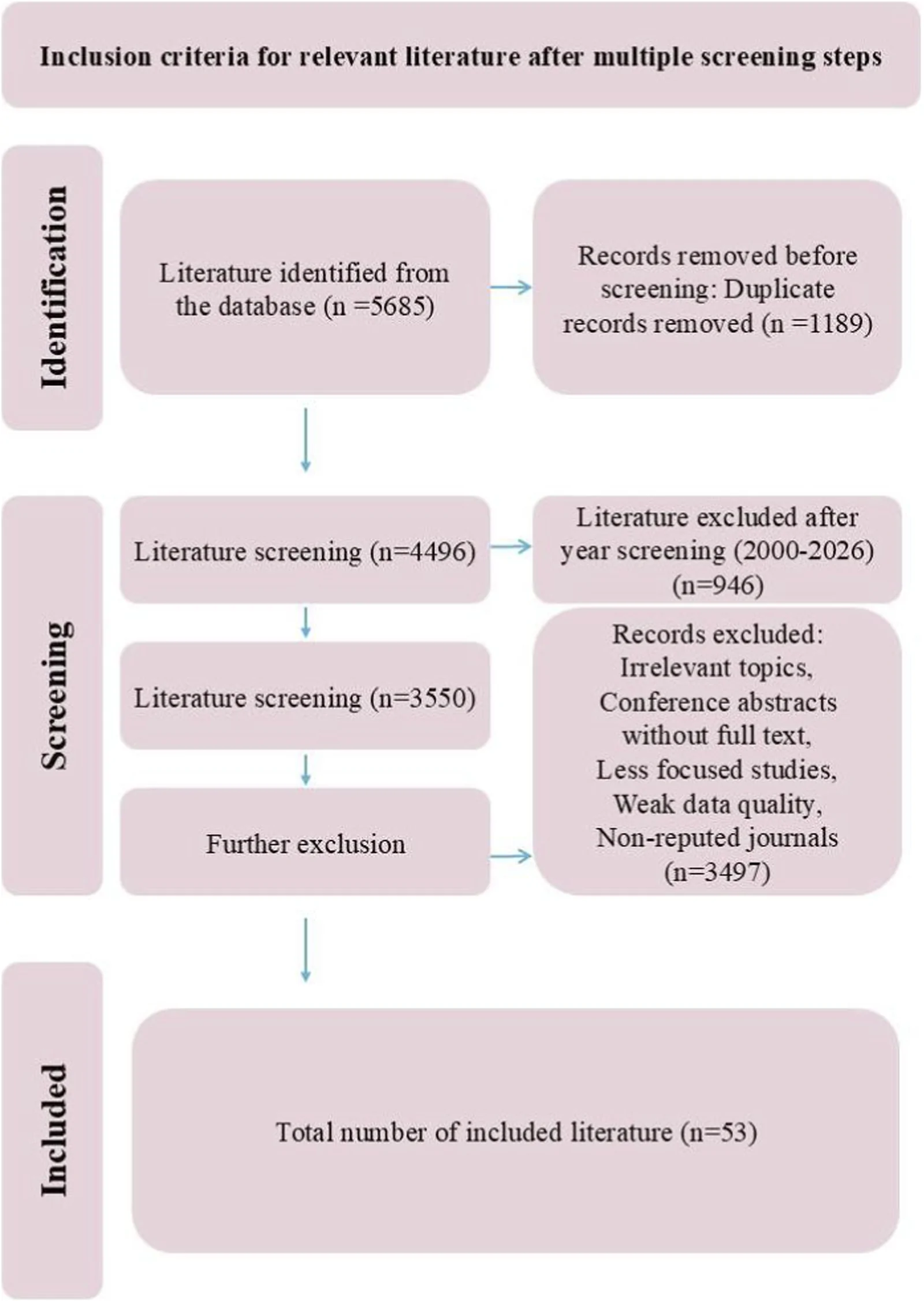
PRISMA flow diagram of the systematic literature search and selection process.

## 3 Preparation of red ginseng polysaccharides

There are many methods for extracting plant polysaccharides, such as hot water extraction (HWE), ultrasound assisted extraction (UAE), microwave-assisted extraction (MAE), enzyme-assisted extraction (EAE), etc (Yang et al., 2025). The commonly used extraction method for red ginseng polysaccharides is HWE (Table 1). The characteristics of this method are simple operation, high security, and low cost. Jin et al. (2021) used HWE to extract polysaccharides (RGPs) from red ginseng. The extraction conditions were extraction time of 5 h, extraction temperature of 85 °C, solid-liquid ratio of 1:20, and polysaccharide yield of about 20%. UAE is also a commonly used method for extracting polysaccharides from red ginseng. Youn et al. (2020) used UAE to extract polysaccharides from red ginseng (RGNP). The extraction conditions were extraction time of 4 h, extraction temperature of 40 °C, and solid-liquid ratio of 1:3. The above steps were repeated 3 times, and the polysaccharide yield was 37.5%. It can be seen that the yield of red ginseng polysaccharides obtained by different extraction methods varies. After reviewing existing literature on red ginseng polysaccharides, it was found that some research teams directly purchase red ginseng concentrate and then use ethanol precipitation to obtain red ginseng polysaccharides. Although preparing red ginseng extract is the most basic experimental operation for extracting polysaccharides. However, red ginseng concentrates products usually have clear quality standards and compared to directly using plant raw materials (which are greatly affected by growth environment, harvesting period, and storage conditions), their batch consistency is better. This is particularly important for polysaccharides, which are highly sensitive to structural integrity, and is conducive to establishing a stable quality control system. In addition, the preparation of polysaccharides based on standardized crude extracts can help improve the reproducibility of experiments and lay the foundation for precise exploration of purification methods.

**TABLE 1:** A summary of the extraction methods of red ginseng polysaccharides.

Name	Extraction method	Time	Temperature	Solid–liquid ratio (g/mL)	Polysaccharide yield (%)	Purification method	References
ELHPP-RGBPs	ELHPP	3 h	Enzyme eaction at 50 °C–60 °C, hot water extraction at 80 °C	1:10	6.91%	Filtration, soaking, and centrifugation	Kim et al. (2019)
FGEP-A	EAE	24 h	90 °C	1:3	1.5%	Dialysis	Song et al. (2018)
FGEP-C	EAE	24 h	50 °C	1:3	2.7%	Dialysis	Song et al. (2018)
FGEP-CA	EAE	24 h + 24 h	The first stage:50 °C The second stage: 90 °C	1:3	2.2%	Dialysis	Song et al. (2018)
FGWP	HWE	4 h × 3	100 °C	1:3	3.2%	Dialysis	Song et al. (2018)
GPR-1 (pectin polysaccharides)	HWE	4 h × 3	100 °C	/	/	DEAE-cellulose column and sephadex G-100 column	Jiao et al. (2014)
GPR-2 (pectin polysaccharides)	HWE	4 h × 3	100 °C	/	/	DEAE-cellulose column and sephadex G-100 column	Jiao et al. (2014)
HPP-RGBPs	HWE	3 h	Before HWE at 80 °C, it is first maintained under a pressure of 550 MPa for 1 min, and then dried at 60 °C	1:10	6.06%	Filtration, soaking, and centrifugation	Kim et al. (2019)
RG0203 (pectin-like polysaccharide)	UAE	1 h	70 °C	1:10	/	Deproteinization, dialysis, and DEAE-cellulose column	Zhang et al. (2025)
RGAP (acidic polysaccharide)	WE	/	/	1:5	/	Dialysis	Choi et al. (2008)
RGAP (acidic polysaccharide)	UAE	4 h × 3	40 °C	1:3	5.5%	DEAE-cellulose chromatography and dialysis	Youn et al. (2020)
RGAP (acidic polysaccharide)	Ethanol extraction, and HWE	Extraction with 70% ethanol: 3 h* 4 times, hot water extraction: 3 h	Ethanol extraction: 70 °C, hot water extraction: 95 °C	1:5	/	Ultrafiltration	Kwak et al. (2010)
RGBPs	HWE	3 h	80 °C	1:10	5.08%	Filtration and centrifugation	Kim et al. (2019)
RGNP (neutral polysaccharide)	UAE	4 h × 3	40 °C	1:3	37.5%	DEAE-cellulose chromatography and dialysis	Youn et al. (2020)
RGPs	HWE	5 h	85 °C	1:20 (w/v)	∼20%	Solid-phase extraction	Jin et al. (2021)
RGP1-1 (Heteropolysaccharide)	WE	2 h	/	1:16	/	DEAE-cellulose chromatography and sephadex G-100 chromatography column	Lian et al. (2022)

Abbreviation: enzyme-assisted extraction (EAE), enzyme-linked high-pressure process (ELHPP), hot water extraction (HWE), ultrasound assisted extraction (UAE), water extraction (WE).

The initially extracted red ginseng polysaccharides generally contain a large number of impurities, such as proteins, pigments, and small molecules, which can affect the purity, biological activity, and stability of the red ginseng polysaccharides (Lu et al., 2025; Yang et al., 2025). In addition, the presence of impurities also poses difficulties for evaluating the structure-activity relationship of polysaccharides. Therefore, it is necessary to further separate and purify it. The Sevage method is commonly used to remove protein from red ginseng polysaccharides (Rao et al., 2024). Lian et al. (2022) obtained red ginseng extract by HWE and then added ethanol precipitation to this solution. Then, protein was removed using Sevage method, small molecules were removed using dialysis method, and finally red ginseng polysaccharide (RGP) was obtained. The commonly used purification methods for red ginseng polysaccharides include centrifugation, dialysis, and column chromatography separation (Figure 3). The commonly used column chromatography separation methods are ion exchange chromatography and size-exclusion chromatography (Jiao et al., 2021; Ren et al., 2023). Overall, although existing research provides references for the extraction and purification of polysaccharides from red ginseng, there are still problems with low extraction rates and single purification methods for red ginseng polysaccharides. In the future, research on the extraction and purification methods of red ginseng polysaccharides should be strengthened to establish a high-yield, high-purity, and highly reproducible red ginseng polysaccharide preparation process, laying the foundation for its structural analysis, activity evaluation, and industrial application, and facilitating its high-value processing and utilization.

**FIGURE 3:**
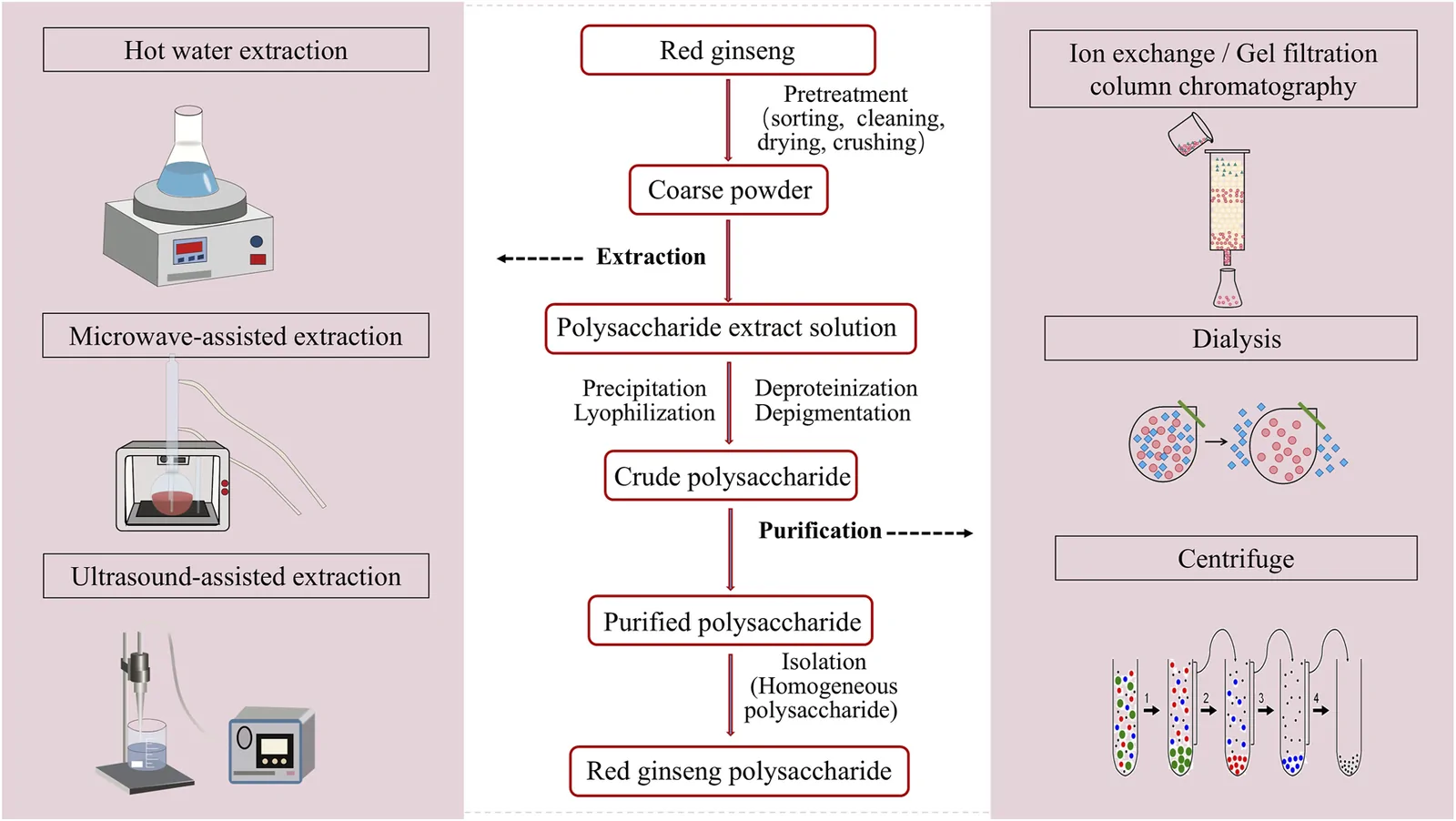
The main extraction and purification steps of red ginseng polysaccharides.

## 4 Structural characteristics of red ginseng polysaccharides

Analyzing the structural characteristics of polysaccharides is a prerequisite for studying their relationship with biological activity. This chapter summarizes the structural characteristics of red ginseng polysaccharides, including monosaccharide composition, molecular weight (Mw), and chemical structure.

### 4.1 Monosaccharide composition

The analysis of monosaccharide composition of polysaccharides is an important step in polysaccharide quality control and obtaining basic information about polysaccharides (Wang et al., 2024). The differences in extraction and purification methods, origin and variety affect the composition of polysaccharides. At present, conventional analytical techniques for red ginseng polysaccharides include high performance liquid chromatography (HPLC), gas chromatography-mass spectrometry (GC-MS), high-performance ion chromatography (HPIC), etc. The monosaccharide composition analysis results showed that red ginseng polysaccharides mainly contain rhamnose (Rha), fucose (Fuc), arabinose (Ara), xylose (Xyl), mannose (Man), galactose (Gal), glucose (Glc), galacturonic acid (GalA), glucuronic acid (GlcA), and fructose (Fru) (Choi et al., 2008; Kwak et al., 2010; Lee et al., 2021) (Table 2). Based on their acidity or alkalinity, polysaccharides can be classified into acidic polysaccharides, neutral polysaccharides and alkaline polysaccharides. Summarizing existing literature, it is found that red ginseng polysaccharides mainly contain acidic polysaccharides and a small number of neutral polysaccharides. RGP-AP-I is an acidic polysaccharide found in red ginseng, with a composition of Rha: Fuc: Ara: Xyl: Man: Gal: Glc: GalA: GlcA= 9.5 ± 0.2: 0.1 ± 0.1: 35.0 ± 0.4: 0.3 ± 0.0: 0.4 ± 0.1: 30.4 ± 0.2: 1.7 ± 0.0: 18.4 ± 1.1: 1.5 ± 0.1 (Lee et al., 2020). Youn et al. (2020) isolated a neutral polysaccharide (RGNP) and an acidic polysaccharide (RGAP) from red ginseng powder through ultrasonic extraction. After processing ginseng into red ginseng, it mainly contains acidic polysaccharides, which is speculated to have changed the tissue structure and polysaccharide form of ginseng during the processing. Further research is needed to determine how the processing process specifically affects the internal structure of ginseng, leading to the high content of acidic polysaccharides in red ginseng.

**TABLE 2:** Monosaccharide compositions of polysaccharides from red ginseng.

Name	Ara	Fru	Fuc	Gal	GalA	Glc	Rha	Ref
FGEP-A	10.9	-	-	12.6	23.1	50.9	1.8	Song et al. (2018)
FGEP-C	10.5	-	-	10.4	14.3	62.2	2.1	Song et al. (2018)
FGEP-CA	11.4	-	-	16.5	22.3	45.8	3.2	Song et al. (2018)
FGWP	10.1	-	-	9.2	17.8	60.6	1.8	Song et al. (2018)
GPR-1	54.57 (%)	0.62 (%)	-	19.96 (%)	10.64 (%)	12.29 (%)	1.92 (%)	Jiao et al. (2014)
GPR-2	8.55 (%)	2.77 (%)	-	18.76 (%)	61.55 (%)	1.52 (%)	6.85 (%)	Jiao et al. (2014)
GPS-1	34.04 (%)	-	-	48.06 (%)	13.31 (%)	3.15 (%)	7.44 (%)	Jiao et al. (2021)
GPS-2	15.29 (%)	-	-	19.23 (%)	55.75 (%)	4.17 (%)	5.56 (%)	Jiao et al. (2021)
RG0203	10.5 (%)	-	1.6 (%)	6.7 (%)	67.5 (%)	4.2 (%)	3.6 (%)	Zhang et al. (2025)
RGP1-1	-	-	-	4.92	-	94.26	-	Lian et al. (2022)

Abbreviation: arabinose (Ara), fucose (Fuc), fructose (Fru), galactose (Gal), galacturonic acid (GalA) glucose (Glc), rhamnose (Rha).

### 4.2 Molecular weight

Polysaccharides are composed of multiple monosaccharides condensed and dehydrated and are a class of complex and massive carbohydrate substances with complex molecular structures (Tao et al., 2025). Obtaining the size and distribution of Mw is of great significance for understanding the structure of polysaccharides. The Mw of polysaccharides is closely related to their physical and chemical properties and directly affects their functional performance in organisms (Luan et al., 2022; Li et al., 2023; Wang et al., 2023). The common determination methods of red ginseng polysaccharide include gel permeation chromatography (GPC), HPLC and static light scattering (SLS). The Mw range of red ginseng polysaccharides is 5655 Da–9.61 × 10^5^ Da. The polysaccharide RGP-AP-I with a Mw of 96 kDa was extracted from red ginseng (Lee et al., 2020). Park et al. (2020) isolated a polysaccharide (KRG-P) from red ginseng and determined its Mw to be 478, 106, and 25 kDa. Lian et al. (2022) extracted and purified a heteropolysaccharide RGP1-1 and determined its Mw to be 5655 Da. Research has found that the effect of the Mw of red ginseng polysaccharides on biological activity is not simply a positive or negative correlation. Based solely on Mw, the activity of red ginseng polysaccharides cannot be predicted. It is necessary to comprehensively consider structural characteristics such as monosaccharide composition and glycosidic bond types.

### 4.3 Chemical structure

The primary and tertiary structures of polysaccharides jointly determine their physicochemical properties and biological activity, and the structural analysis of polysaccharides plays a very important role in polysaccharide research (Zeng et al., 2019; Zhu et al., 2025). Elucidating structural information is necessary to clarify the specific recognition between polysaccharides and receptors, reveal signal transduction pathways, and guide targeted modifications. For example, the triple helix conformation of *β*-1,3-glucan is crucial for its binding to Dectin-1 and activation of NF-κB. Once the branched chain is disrupted, the immune enhancing activity sharply decreases. After clarifying the structure, precise functional modifications can be made to target the corresponding biological activity while maintaining the integrity of the main chain (Meng et al., 2020; Guo et al., 2021). In addition, in-depth study of the chemical structure of polysaccharides lays the foundation for establishing their structure-activity relationship (Kou et al., 2024; Luan et al., 2024). This is a complex and important task. This chapter summarizes the existing research on the chemical structure of red ginseng polysaccharides. Lee et al. (2020) extracted a red ginseng polysaccharide RGP-AP-I. Methylation analysis showed that 21 different glycosidic bond linkage modes, including 3-, 4-, 6-, and 3,6-linked Gal*p*, 5-linked Ara*f*, 2,4-dnected Rha*p*, and 4-linked GalA*p*. In addition, the reaction results of the beglucosyl Yariv reagent indicate that RGP-AP-I contains arabino-β-3,6-galactan. Furthermore, the team used four specific glycosidases for gradient enzymatic hydrolysis and identified the fine structure of RGP-AP-I using *β*-elimination chemical decomposition technology and various chromatographic and spectroscopic analysis techniques. The results showed that RGP-AP-I is based on the rhamnogalacturonan I skeleton, consisting of repeated disaccharide units [→2)-Rha*p*-(1 → 4)-GalA*p*-(1→] and three side chains substituted at the C(O)4 position of the rhamnose residue in the backbone. After identification, these three side chains are highly branched *α*-(1→5)-arabinan, a branched *β*-(1→4)-galactan, and an arabino-*β*-3,6-galactan (Lee et al., 2021) (Figure 4). Lian et al. (2022) purified a heteropolysaccharide (RGP1-1) by column chromatography and analyzed its chemical structure using methylation and NMR. The results indicate that RGP1-1 consists of a main chain composed of → 1)-Glc*p* (4 → and →1)-Galp-(4 →, branching at the O-4 position, with the branching group being *α* -D-Glc*p*-(1→.

**FIGURE 4:**
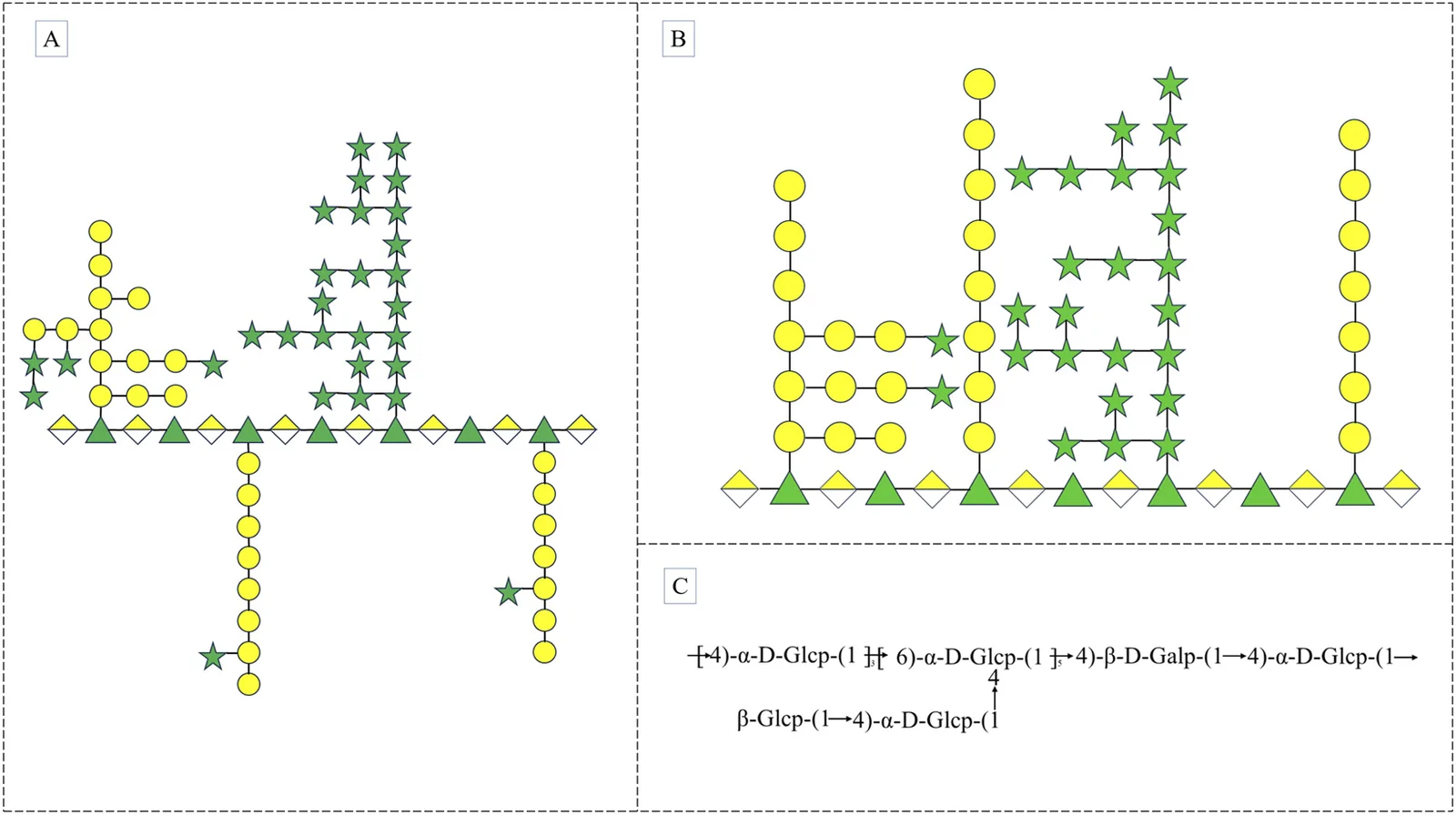
The speculated structure of polysaccharides in red ginseng **(A)** RGP-AP, **(B)** RGP-AP-I, **(C)** RGP1-1.

## 5 Immunomodulatory effect of red ginseng polysaccharides

The immune system is an important system in the body that plays a role in immune function. It can resist pathogen invasion, coordinate with other systems in the body, and jointly maintain physiological functions and relative homeostasis of the internal environment (Ying and Hao, 2023). Research has shown that polysaccharides from red ginseng have immunomodulatory effects, which can enhance immunity through various pathways such as affecting immune cell activity, regulating cytokine levels, and promoting immune organ development (Figure 5).

**FIGURE 5:**
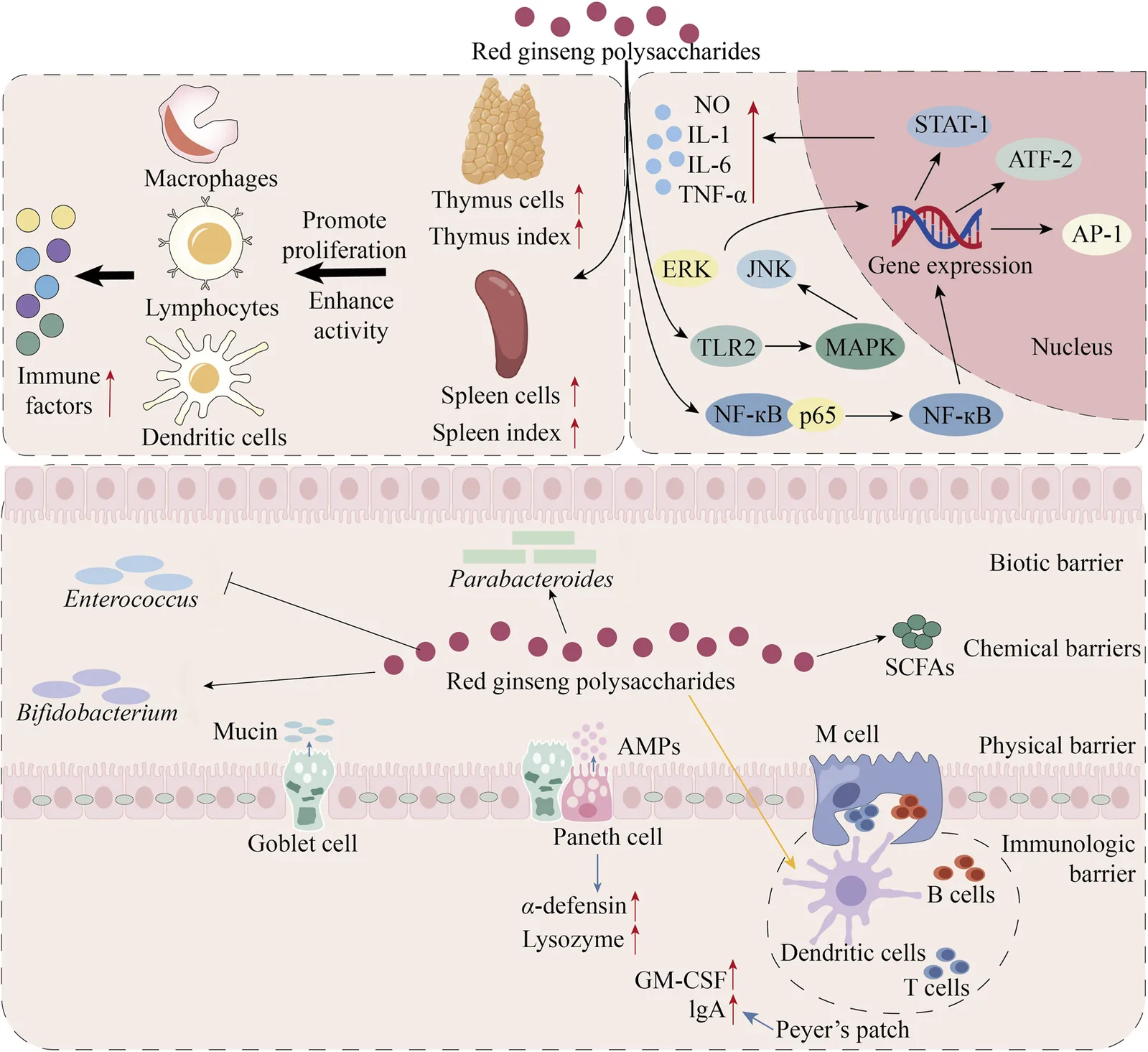
The potential mechanism of the immunomodulatory effect of red ginseng polysaccharides.

Byeon et al. (2012) found through *in vitro* experiments that red ginseng polysaccharides (RGAP) treatment (0–4 mg/mL) can induce the production of nitric oxide (NO) in RAW264.7 cells, but does not cause morphological changes, which is different from lipopolysaccharide (LPS). To confirm whether RGAP induced NO production is regulated by activation of cellular transcription factors, the expression level of inducible nitric oxide synthase (iNOS) and the transcription factors required for iNOS were subsequently detected. The results showed that RGAP induced an increase in iNOS mRNA levels, but lower than the LPS group. Increased nuclear translocation levels of NF-κB (p65), AP-1 (c-Jun and c-Fos), CREB, ATF-2, and STAT-1 were also observed in the RGAP treatment group. It is speculated that RGAP induced NO production is related to the increase in iNOS levels and nuclear transcription factor levels. Evaluation through the use of specific enzyme inhibitors, Western blot analysis of intracellular signaling proteins, and inhibition patterns of antagonistic antibodies indicates that ERK and JNK are the most important signaling enzymes of RGAP, and TLR2 may be the surface receptor of RGAP. Finally, macrophages isolated from RGS2 gene knockout mice or cells treated with wortmannin showed a significant increase in the production of NO. Therefore, it is speculated that RGAP can activate the function of macrophages and exert immune effects by activating transcription factors and their upstream signaling enzymes (such as ERK and JNK). In addition, Lee et al. (2020) demonstrated the immunostimulatory activity of red ginseng polysaccharides (RGP-AP-I) through indicators such as anti-complement activity and macrophage stimulatory activity. The Peyer’s patch cell in the mucosal lymphoid tissue of the small intestine contains many immune cells and are the main source of IgA in the body. IgA is an important class of antibodies in the human immune system, playing a central role in resisting pathogen invasion and maintaining mucosal tissue health. Park et al. (2020) studied the stimulatory activity of red ginseng polysaccharides (KRG-P) at different concentrations on Peyer’s patch cells, with lipopolysaccharides as a positive control. After treatment with KRG-P (at concentrations of 125, 250, or 500 μg/mL), Peyer cells secrete granulocyte macrophage colony-stimulating factor (GM-CSF) in a concentration dependent manner. After treatment with 500 μg/mL KRG-P, the secretion of IgA by Peyer’s patch cells significantly increased (*P* < 0.05).

Other research groups have investigated the immunomodulatory effects of red ginseng polysaccharides through *in vivo* experiments. Youn et al. (2020) extracted two types of polysaccharides, namely neutral polysaccharides (RGNP) and acidic polysaccharides (RGAP). To compare the immune activity of different components, 40 mice were randomly divided into 5 groups: normal group, cyclophosphamide (CY) control group, water fraction (W) group, RGNP group, and RGAP group. Then, continue to administer for 10 days (100 mg/kg). CY was administered 5 days before autopsy, followed by measurement of immune organ weight. In all groups given CY, the relative and absolute weights of immune organs were significantly reduced (*P* < 0.05). Compared with the CY control, administration of W, RGNP, and RGAP induced recovery of relative and absolute weight of spleen and thymus, but without significant changes. Furthermore, the ability of different components to form antibody-forming cells (AFC) in animals with weakened immunity was compared, and it was found that the RGAP component formed the highest number of AFC. To determine the optimal dosage of the RGAP component with the highest immune activity, macrophage activity and spleen cell subtypes were analyzed. Compared with the CY group, the phagocytic activity of macrophages significantly increased in a dose-dependent manner after administration of 50, 100, and 200 mg/kg RGAP (*P* < 0.05, *P* < 0.01, *P* < 0.01). In addition, the number of T cells, B cells, and macrophages significantly increased in the RGAP (100, 200 mg/kg) group. These results indicate that RGAP has an enhanced immune function (Youn et al., 2020). These results not only provide scientific evidence of the traditional efficacy of red ginseng but also offer inspiration and reference for its further development and application.

The intestine can digest and absorb nutrients and undergo metabolism, as well as resist pathogen invasion, making it an important site for mucosal immunity to function (Xie et al., 2025; Yu et al., 2025; Wu et al., 2023). Park et al. (2020) investigated the role of KRG-P in regulating intestinal immunity *in vivo*. Mice were orally administered KRG-P (5 mg/kg and 50 mg/kg) daily for 10 days. On the 6th and 11th day after administration, the secretion and mRNA expression levels of IgA in feces were measured. After the complete administration, the protein content of *α*-defensin-1 in the intestinal tissue of mice was also measured. On the 6th day, the production of IgA increased in all treatment groups. On the 11th day, the secretory IgA levels in the KRG-P treatment group significantly increased in a dose-dependent manner. Measurement of IgA mRNA expression levels in intestinal tissue revealed a significant increase (*P* < 0.05, *P* < 0.01) after treatment with KRG-P (5 mg/kg and 50 mg/kg). Compared with the normal group, treatment with 50 mg/kg KRG-P significantly increased the mRNA and protein levels of *α*-defensin-1 in the intestinal tissue of mice (Park et al., 2020). It can be inferred that KRG-P has the potential to regulate intestinal immunity. Subsequently, the team further investigated the effects of oral administration of KRG-P (50 mg/kg or 200 mg/kg) to mice on the mRNA and protein expression levels of *α*-resistin, lysozyme, and E-cadherin in the small intestine, as well as changes in the content of SCFAs in the cecum. The group treated with KRG-P enhanced the mRNA and protein expression of *α*-resistin and lysozyme in the small intestine tissue. In addition, the increased expression of E-cadherin strengthens the intestinal barrier and improves the content of short chain fatty acids in the cecum (Kim et al., 2024).

Based on these findings, the team evaluated the effectiveness of KRG-P in alleviating diarrhea using a model triggered by antibiotic-associated diarrhea (AAD). Observing the weight, diarrhea status, and water intake of mice, it was found that compared with the AAD group, after KRG-P treatment (100 mg/kg and 300 mg/kg), the weight of mice recovered, diarrhea symptoms were relieved, and water intake returned to normal. The use of KRG-P also increased the protein and mRNA expression levels of claudin-1 and lysozyme. The relationship between gut microbiota and diarrhea is close, and dysbiosis of the microbiota can directly induce or worsen diarrhea, which can further damage the gut microbiota. The proportion of *Bacteroidetes* and *Firmicutes* in the classification of gut microbiota is used as an indicator to measure the health status of the body (Zhu et al., 2024). The analysis of changes in the gut microbiota of mice showed a decrease in *Firmicutes* and *Bacteroidetes* abundance in the AAD group. However, after administering KRG-P, the richness of the above two bacteria increased. In addition, analysis of SCFAs in the cecum showed a significant decrease in the content of SCFAs in the AAD group, but showed an increasing trend after administration of KRG-P. It can be inferred that KRG-P can improve diarrhea caused by lincomycin (Min et al., 2024).

The immunomodulatory effect of red ginseng polysaccharides may be achieved by directly activating macrophages and dendritic cells through pattern recognition receptors such as TLR2. This mechanism has been preliminarily validated in *in vitro* cell experiments. On the other hand, red ginseng polysaccharides can regulate intestinal immunity through Peyer’s patches. In the AAD model, it can also restore the balance of gut microbiota, increase the content of cecal SCFAs, and upregulate the expression of tight junction proteins claudin-1 and lysozyme. It should be pointed out that this study did not involve the specific microbial degradation process of red ginseng polysaccharides in the intestine, detection of short chain fatty acids, and evaluation of intestinal barrier function. Therefore, although existing results support the regulatory effect of red ginseng polysaccharides on local gut microbiota and barriers, it is not yet possible to confirm their role through the “microbiota, metabolite barrier, immune” axis. In the future, the relative contribution of indirect pathways and their synergistic relationship with direct pathways need to be further verified through the above experimental design.

## 6 Immunomodulatory effect of red ginseng polysaccharides combined with other drugs

The combination of red ginseng polysaccharide (RGAP) and other drugs also has immunomodulatory effects. In the CY induced immunosuppressive animal model, the combination of RGAP and pidotimod showed synergistic immunomodulatory activity. CY reduced NK cell activity to 59.4%. Compared with the blank control group, CY also caused a decrease in mice spleen proliferation ability. Both pidotimod and RGAP, when used alone or in combination, can significantly enhance the proliferation ability of damaged mice spleen T cells and the phagocytic ability of NK cells. However, the combination of pidotimod and RGAP (300 mg/kg) is necessary to restore the proliferation ability of splenic B cells. NO mediates cytotoxicity in the immune system, participating in the killing of microorganisms, protozoa, and tumor cells. The combination of pyridomode (200 mg/kg) and different doses of RGAP (100 mg/kg and 300 mg/kg) significantly increased the production of NO to 86.1% and 87.1%, respectively, compared to the single treatment. The levels of IL-12, IFN-γ, and C-reactive protein (CRP) in mice serum were evaluated, and it was found that CY caused a decrease in these cytokines and CRP levels. The combination of two drugs can significantly increase serum IL-12 and IFN-γ levels. RGAP alone or in combination with pidotimod can regulate serum CRP levels to near normal levels (Du et al., 2008a). It can be inferred that RGAP and pidotimod can enhance the proliferation and vitality of immune cells, promote the release of immune regulatory factors, and achieve the effect of improving cellular immune levels.

Subsequently, the team also studied the effects of pidotimo and RGAP on humoral immunity. Use plaque-forming cell (PFC) assay to detect the immune response of thymus-independent antigen (TI-Ag). LPS in mice, and at the same time detect the changes in IgM production in mice serum. RGAP alone or in combination with pidotimod effectively resisted the decrease in serum IgM levels caused by immunosuppressants (methotrexate). However, the combination of pidotimod and RGAP (300 mg/kg) significantly increased the PFC count of splenic cells, which was 2.4 times higher than that of the immunosuppressive group. B cells bind to the antigenic determinants on the sheep red blood cells (SRBC) membrane, and the primary immune response mainly produces IgM, leading to the dissolution of SRBC and the formation of plaques. The secondary response to SRBC relies on the secretion of specific IgG by memory cells generated by the primary immune response. The combination of RGAP and pidotimod can significantly alleviate the leukopenia and splenomegaly caused by CY and enhance the primary and secondary immune responses induced by SRBC. In addition, blood biochemistry and pathological section examination of immune organs showed that the combination of pidotimod and RGAP did not cause liver and kidney damage, and there were no significant pathological changes in the main immune organs and tissues. These results indicate that the combination therapy of pidotimod and RGAP synergistically enhances antibody response to LPS and SRBC stimulation without causing any toxic changes (Du et al., 2008b). The above studies investigated the immunomodulatory effects of pidotimod and RGAP on immunocompromised mice from the perspectives of cellular immunity and humoral immunity and preliminarily revealed the potential synergistic mechanism of the two in restoring immune function. Whether used alone or in combination with chemical immunomodulators, RGAP has shown promising application prospects. It is recommended to conduct more research and clinical trials on the basis of clarifying the optimal effective dose of RGAP in subsequent studies, to verify the effectiveness and safety of its monotherapy and combination therapy and promote the transformation of red ginseng polysaccharides from basic research to clinical immunotherapy.

## 7 Other pharmacological activities of red ginseng polysaccharides

### 7.1 Anti-diabetic effect

Diabetes mellitus (DM) is primarily classified into three types: type 1, type 2, and gestational diabetes. Research has shown that red ginseng polysaccharides can treat type 2 diabetes (T2DM). Yang et al. (2025) explored the therapeutic effect and potential mechanism of red ginseng polysaccharide (RGAP) on T2DM from the perspective of fecal fatty acid (FA) regulation. After the successful establishment of the model, the level of fasting blood glucose (FBG) in diabetes rats increased significantly. After daily gavage administration of RGAP (100 mg/kg/day) for 4 consecutive weeks, FBG levels significantly decreased compared to the model group (*P* < 0.05). Fatty acids are the main components of lipids, and studies have shown that they play an important role in the pathogenesis of type 2 diabetes. Through 2-Dimethylaminoethylamine (DMED) derivatization-based LC-MS method, it was found that the T2DM model affects the composition or concentration of FA in feces, except for C6:0. After RGAP treatment, the strength of most fatty acids recovered and reversed their changes. RGAP treatment significantly upregulated the intensity of C4:0 and C5:0 (*P* < 0.001, *P* < 0.01), while the intensity of C16:0 and C18:0 was downregulated. SCFA intensity is negatively correlated with fasting blood glucose levels, while long-chain fatty acid intensity is positively correlated with fasting blood glucose levels. The above results indicate that RGAP can regulate FA metabolism and has a hypoglycemic effect. In addition, by constructing a metabolite enzyme gene network, it can be inferred that the mechanism of action of RGAP on T2DM may be related to the regulation of fatty acid metabolism and inflammation related signaling pathways (Wang et al., 2024). At present, there are relatively few studies on the anti-diabetes effect of red ginseng polysaccharide, and the existing studies have only studied from the perspective of FA metabolism. In view of the complexity of the pathogenesis of diabetes, its mechanism can be further explored from multiple dimensions such as oxidative stress, intestinal flora and insulin signal transduction to systematically build the pharmacological effect network of red ginseng polysaccharide.

### 7.2 Anti-Alzheimer’s disease effect

Alzheimer’s disease (AD) is the most common neurodegenerative disease. In recent years, some studies have shown that red ginseng polysaccharides have the potential to treat or improve AD. Shin et al. (2021) investigated the therapeutic effect of non-saponin fraction with rich polysaccharide (NFP) in red ginseng on AD (Figure 6). Firstly, the effect of NFP on proteins in the brain of aged rats was analyzed using proteomics. The results indicate that NFP helps improve the structure and function of central nervous system tissues and may have a protective effect on brain tissue. Then, the therapeutic effect of NFP on AD was studied using 5XFAD and wild type (WT) mice at 5.5 months of age. Immunofluorescence staining with 4G8 antibody was performed on the subiculum of 5XFAD mice, and it was found that compared with vehicle-treated 5XFAD mice, the area fraction, average size, and Aβ deposition of 5XFAD mice treated with NFP were significantly reduced (*P* < 0.001). Inflammatory response plays a crucial role in the occurrence and development of AD (Botella and Heneka, 2024). To further investigate the anti-inflammatory effect of NFP treatment, immunohistochemistry staining was performed on the subiculum of 5XFAD mice using Iba-1 microglial cell marker. It was found that the Iba-1 (+) region of 5XFAD mice treated with NFP was significantly reduced compared to vehicle created 5XFAD mice (*P* < 0.001). Evaluate the neuroprotective effect of NFP treatment on the subiculum of 5XFAD mice using NeuN antibody through immunofluorescence staining. The results showed that the number of NeuN positive cells in vehicle-treated 5XFAD mice was significantly lower than that in vehicle-treated WT mice (*P* < 0.001), while the number of NeuN positive cells in NFP treated 5XFAD mice was significantly higher than that in vehicle-treated 5XFAD mice (*P* < 0.001). To evaluate the effect of NFP on mitochondrial dynamics in AD brain, immunofluorescence staining was also performed on the pubic tissue of 5XFAD mice using antibodies targeting the mitochondrial outer membrane marker translocases of mitochondrial outer membrane 20 (Tom20). Compared with vehicle-treated 5XFAD mice, 5XFAD mice treated with NFP showed a significant improvement in mitochondrial quantity and an increase in mitochondrial fusion. The NFP treated 5XFAD mice showed fragmentation similar to that of vehicletreated WT mice. Overall, NFP can significantly alleviate AD related pathological phenomena, including Aβ deposition, neuroinflammation, neurodegenerative disorders, and mitochondrial dysfunction. In addition, the effect of NFP on Aβ-mediated mitochondrial respiratory defects in HT22 mouse hippocampal neuronal cells (HT22) was analyzed using Seahorse XFp analysis. NFP can prevent neuronal dysfunction caused by Aβ and cell apoptosis caused by aging by reducing mitochondrial dysfunction. Moreover, NFP treatment significantly increased adult hippocampal neurogenesis (AHN) and neurite outgrowth of neural stem cells in healthy brains and AD brains. Finally, the Y-maze test was used to observe the effect of NFP on cognitive impairment in Aβ-induced 5XFAD AD mice. NFP administration significantly enhanced and restored cognitive function in healthy and AD mice. The above suggests that NFP may be one of the effective drugs for anti-aging and treating AD (Shin et al., 2021).

**FIGURE 6:**
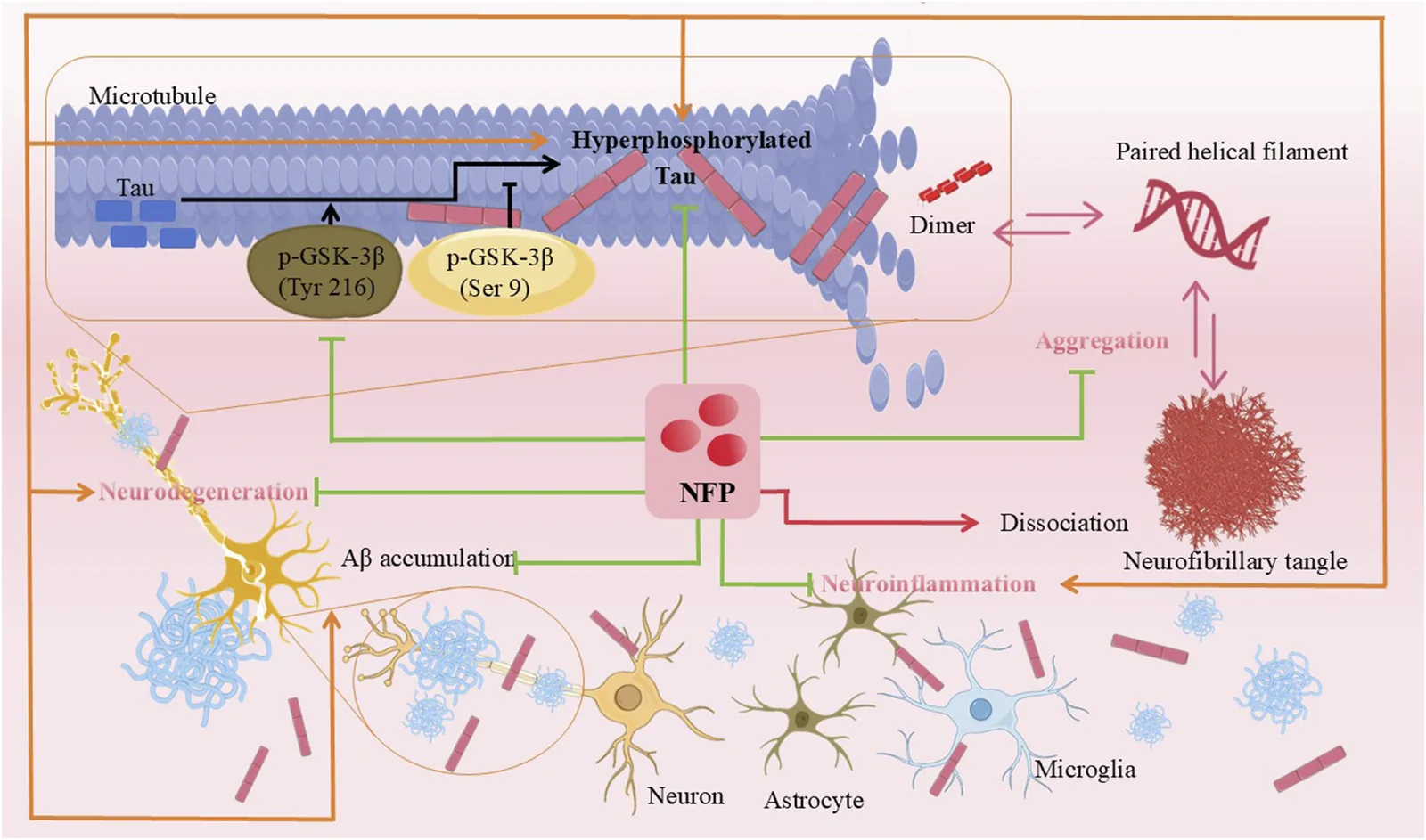
The potential mechanism of non-saponin fraction with rich polysaccharide in the treatment of Alzheimer’s disease.

Subsequently, the team further investigated the molecular mechanism of NFP’s therapeutic effect on AD using proteomics. According to research findings, it is speculated that the therapeutic effect of NFP on AD is related to synaptic and mitochondrial pathways (Kim et al., 2023). In addition, the team also studied the effects of NFP on various tau lesions using *in vitro* (HT22) and *in vivo* (triple transgenic (3xTg) mice) models. Tau protein is closely related to the progression of neurodegenerative diseases Continuous administration of NFP (200 mg/kg/d) for 3 weeks inhibited the aggregation and dissociation of tau aggregates formed *in vitro*, and significantly reduced tau protein hyperphosphorylation induced by Okada acid and hippocampal neuron loss caused by tau protein. In addition, treatment with NFP can improve the accumulation of hyperphosphorylated tau protein and tau tangles in the brains of 3xTg mice, as well as pathological phenomena associated with AD (Kim et al., 2024). It is worth noting that NFP, as an enriched component of the non-saponin part of red ginseng, has an acidic polysaccharide content of up to 438.08 mg/g, indicating that polysaccharides are likely the main material basis for the anti-AD effect of this component. However, due to the possible coexistence of other components in NFP, it is currently not possible to fully attribute all observed anti-AD effect to polysaccharides. This limitation precisely suggests that future research needs to further isolate and purify NFP to obtain homogeneous polysaccharide. To clarify whether red ginseng polysaccharides themselves are sufficient to reproduce all or part of the pharmacological activity of NFP.

### 7.3 The health benefits for the skin

The skin serves as a vital barrier and organ of the body, protecting against pathogens, ultraviolet (UV) radiation, and pollution while regulating body temperature, retaining moisture, and sensing external stimuli. Therefore, daily skincare and timely repair after damage are crucial (Li et al., 2025). However, factors such as UV radiation, oxidative stress, and atopic dermatitis in daily environment continuously impair the skin’s barrier function, leading to frequent issues like aging, sensitivity, and itching. Research indicates that red ginseng polysaccharides (ELHPP-RGBPs) possess protective effects against the skin, including antioxidant, anti-aging, and anti-atopic dermatitis properties (Kim et al., 2019). In acute toxicity tests and skin irritation tests, no toxic symptoms or adverse reactions were observed after treatment with red ginseng polysaccharides (2,000 mg/kg). The ABTS radical scavenging test results indicated that the positive control Trolox (1,000 μg/mL) exhibited an antioxidant activity of 43.33 ± 1.15 μM, while ELHPP-RGBPs (10,000 μg/mL) demonstrated an antioxidant activity of 141.7 ± 6.98 μM. Additionally, the researchers examined the anti-aging effects of red ginseng polysaccharides using a UVA-irradiated HaCaT cell model. ELHPP-RGBPs reduced sUV-induced MMP-1 expression without affecting MMP-2. Compared to the MMP-1 mRNA levels in normal HaCaT cells, sUV-induced MMP-1 mRNA levels increased by up to 216%. When treated with 20 μg/mL ELHPP-RGBP, this increase was reduced to 117%. Similarly, AP-1 transcriptional activation decreased from 203% to 143% with 20 μg/mL ELHPP-RGBP treatment. These findings suggest that ELHPP-RGBPs may have anti-aging potential. Beyond this, skin lesion analysis, dermatitis scoring, and skin thickness assessments revealed that ELHPP-RGBPs alleviated symptoms resembling atopic dermatitis induced by dermatophagoides farinae extract. These experiments provide scientific references for the application of red ginseng polysaccharides as cosmetic ingredients. It is particularly important to note that red ginseng polysaccharides (RGAP) can promote the expression of inflammation-related biomarkers such as IL-1*β*, IL-6, IL-8, and TNF-*α* in sebaceous gland cell and outer root sheath (ORS) cell cultures. After LPS stimulation, the inflammatory response to *Cutibacterium acnes* and the resulting nodules in mice were more severe when treated with RGAP. Based on this experiment, it is speculated that products containing red ginseng polysaccharides may not be suitable for patients with inflammatory acne (Lee et al., 2021). However, the above results still need to be further confirmed through more in-depth *in vivo* experiments and clinical data.

### 7.4 Anticancer effect

Cancer is the general term for malignant tumors, characterized by invasiveness and metastasis, posing a serious threat to human health and ranking as one of the leading causes of death worldwide (Lai et al., 2024). Existing studies have shown that red ginseng polysaccharides exhibit anticancer effect in both cellular and animal models. Zhai et al. (2022) selected lung cancer cell line A549 and triple-negative breast cancer cell line MDA-MB-231 to investigate the anticancer effects of RGP (red ginseng polysaccharides) *in vitro*. RGP inhibited the proliferation of human A549 and MDA-MB-231 cells, with IC_50_ values of 376.2 μmol/L and 311.3 μmol/L, respectively. Additionally, RGP induced lactate dehydrogenase (LDH) release, promoted ferroptosis, and suppressed GPX4 expression. Further reversal experiments were designed, combining RGP treatment at 200 μg/mL with ferroptosis inducers or inhibitors. The effects of RGP enhanced the efficacy of the ferroptosis inducer (erastin) but were eliminated by the ferroptosis inhibitor (ferrostatin-1) (Zhai et al., 2022). Although the team confirmed the anticancer effects of red ginseng polysaccharides, their study relied solely on a single *in vitro* model, and the criteria for assessing ferroptosis were incomplete. Subsequently, other research teams also investigated the anticancer effects of red ginseng polysaccharides; Wang et al. (2024) cultured AGS cells and then divided them into groups treated with different concentrations of RGP (50 μg/mL, 100 μg/mL, and 200 μg/mL). After 48 h, cell viability and apoptosis rates were measured. As the RGP concentration increased, the proliferation level of AGS cells significantly decreased compared to the control group treated with 0 μg/mL RGP. Flow cytometry was used to observe the effect of RGP on AGS cell apoptosis, revealing that the apoptosis level of AGS cells increased with higher RGP concentrations. Therefore, it can be concluded that RGP significantly inhibits AGS cell proliferation and viability while promoting apoptosis. Ferroptosis is a reactive oxygen species (ROS) dependent form of cell death primarily associated with iron accumulation and lipid peroxidation, two biochemical characteristics. After RGP treatment, the levels of ROS, Fe^2+^, malondialdehyde (MDA), and LDH in AGS cells significantly increased. Western blotting further examined the effects of RGP on the expression levels of SLC7A11, GPX4, and ACSL4, confirming that RGP is a key inducer of ferroptosis in gastric cancer. Phosphatidylinositol 3-kinase (PI3K) is an intracellular phosphatidylinositol kinase and a necessary gene for the activation of protein kinase B (Akt). The PI3K/Akt signaling pathway plays a crucial role in physiological processes such as cell proliferation, differentiation, and apoptosis (Fontana et al., 2024). With increasing RGP concentration, the levels of p-PI3K and p-Akt significantly decreased, and the ratios of p-PI3K/PI3K and p-Akt/Akt gradually declined (*P* < 0.01). Aquaporin 3 (AQP3) is a key molecule in cancer therapy, and its abnormal expression is associated with various cancer-related processes. The inhibitory effect of RGP on gastric cancer cells may, to some extent, be achieved by downregulating AQP3 expression. Finally, *in vivo* experiments also demonstrated the inhibitory effect of RGP on gastric cancer cells. In summary, red ginseng polysaccharides promote ferroptosis in gastric cancer cells, and their mechanism may involve downregulating AQP3 to inhibit the activation of the PI3K-Akt signaling pathway (Wang et al., 2024). Based on the evaluation of existing literature, *in vitro* experiments on the anticancer effects of red ginseng polysaccharides are the most abundant, followed by animal model studies, while clinical research is almost blank. How to move from mechanism discovery to reliable clinical validation remains an important challenge in this field.

### 7.5 Cardioprotective effect

The search for safe and effective cardioprotective substances has always been a crucial topic in the fields of medicine and life sciences. Lian et al. (2022) employed an isoproterenol (ISO)-induced myocardial ischemia (MI) animal model to evaluate the protective effects of RGP1-1 against myocardial infarction. Mice pretreated with RGP1-1 exhibited a significant reduction in the cardiac index (*P* < 0.01) compared to the model group. Relative to the blank control group, RGP1-1 groups (100, 200, and 400 mg/kg) showed a marked decrease in myocardial infarction area, reaching 23.54% ± 1.78%, 31.54% ± 3.03%, and 40.09% ± 2.91%, respectively. Biochemical serum marker analysis revealed that RGP1-1 could maintain cell membrane integrity and permeability. When excessive oxygen free radicals cannot be promptly cleared by the body, they attack various cardiac structures through “oxidative stress”. To elucidate RGP1-1 protective effects against MI in mice, this study also assessed its antioxidant activity. RGP1-1 mitigated MI by downregulating ROS and malondialdehyde (MDA) levels in myocardial tissue while increasing SOD, CAT, and GPx levels, thereby suppressing oxidative stress. After MI onset, the body produces large amounts of inflammatory mediators and chemokines, activating various inflammatory pathways and further inducing inflammatory responses. Compared to the model group, the RGP1-1 treatment group showed significantly lower serum levels of inflammatory factors TNF-*α* and IL-6 (*P* < 0.05 or *P* < 0.01). TUNEL assay evaluation of RGP1-1 impact on cardiomyocyte apoptosis revealed a significant reduction in TUNEL-positive cells in the RGP1-1-treated group compared to the model group, similar to the positive control group (verapamil). HE staining observations demonstrated that RGP1-1 reduced cardiomyocyte apoptosis and myocardial fibrosis in mice. Western blot analysis indicated that RGP1-1 upregulates the expression of key protein Nuclear factor E2-related factor 2 (Nrf2), NAD(P)H: quinone oxidoreductase 1 (NQO1), heme oxygenase-1(HO-1) and kelch-like ECH-associated protein1 (keap1) in oxidative stress injure progress, and thereby modulating the Nrf2/HO-1 pathway (Lian et al., 2022) (Figure 7). Furthermore, Zhang et al. (2025) evaluated red ginseng polysaccharide (RG0203) through cell testing and an *in vitro* MI model, and the results showed that RG0203 significantly improved cardiac function. Specifically, at a concentration of 50 μg/mL, the polysaccharide effectively alleviated myocardial cell inflammatory injury induced by oxygen-glucose deprivation (OGD) and hydrogen peroxide (H_2_O_2_). At a concentration of 200 μg/mL, it significantly increased the left ventricular pressure change rate in rats with acute MI. The above results indicate that the prepared polysaccharide RG0203 has potential protective effects against acute MI (Zhang et al., 2025). Another study by the team showed that red ginseng polysaccharides (RG) can enhance mitochondrial function and inhibit damage to H9c2 cells by OGD or oxygen glucose deprived/reperfusion (OGD/R) (Liu et al., 2022). The above findings collectively suggest that the cardioprotective effect of red ginseng polysaccharides may not rely on a single target but may be achieved through a multi axis synergistic network of “mitochondrial energy metabolism-oxidative stress-inflammation regulation”.

**FIGURE 7:**
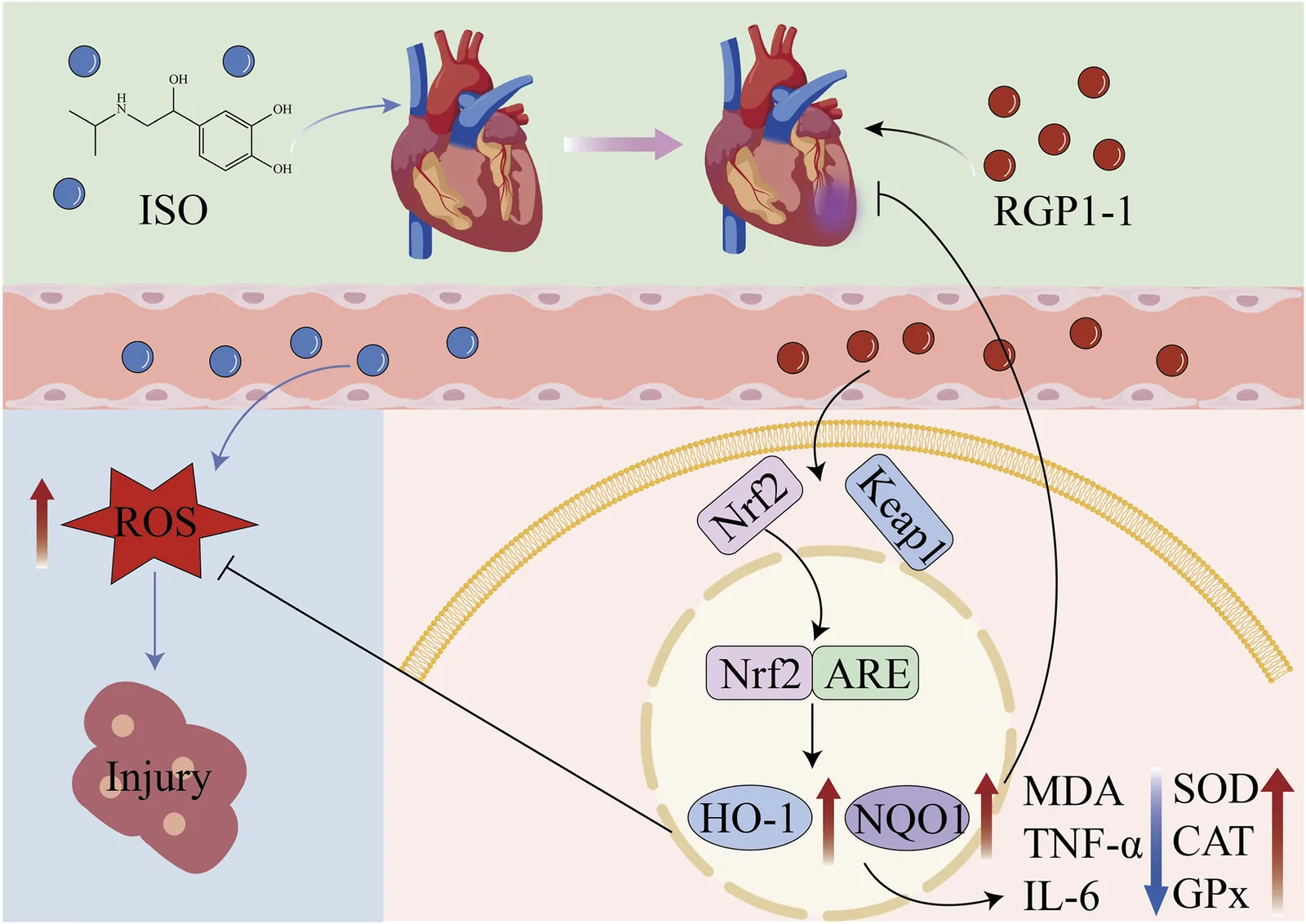
The potential mechanism of the cardioprotective effect of red ginseng polysaccharides.

Although the role of red ginseng in cardiovascular disease has been recognized, research specifically focusing on the cardioprotective effects of red ginseng polysaccharides is still very limited. Especially, clinical trial data on the cardioprotective effect of red ginseng polysaccharides are extremely scarce. The existing research is mostly based on animal and cell experiments, which are not sufficient to support its clinical application as a therapeutic drug for cardiovascular diseases. Therefore, the cardioprotective activity of red ginseng polysaccharides should be understood as preclinical therapeutic potential, and their effectiveness and safety in clinical translation need further verification.

## 8 Structure-activity relationship of red ginseng polysaccharides

Extensive research has attempted to elucidate the correspondence between the chemical structure of polysaccharides and their biological activities. Although the mechanisms are not yet fully understood, parameters such as Mw, monosaccharide composition, glycosidic bond type, branching structure, and chain conformation are known to influence polysaccharide activity (Pan et al., 2024). FGEP-CA is a polysaccharide obtained from red ginseng, with a monosaccharide composition ratio of Rha: Ara: Gal: Glc: GalA=3.2:11.4:16.5:45.8:22.3. The monosaccharide composition of polysaccharides FGWP and FGEP-CA obtained from red ginseng is the same, but the molar ratio is different, which is 1.8: 10.1: 9.2: 60.6: 17.8. Evaluating the effects of FGWP and FGEP-CA on macrophage cytokine production, it was found that compared with the FGWP group, the concentrations of cytokines IL-6, IL-12, and TNF-*α* in FGEP-CA were significantly increased (*P* < 0.05). FGEP-CA exhibits significant immunostimulatory properties *in vitro* (Song et al., 2018). It is speculated that these differences may be caused by differences in their monosaccharide ratios. Mw is also an important factor affecting the biological activity of red ginseng polysaccharides. The red ginseng polysaccharide (GPS-1) with a Mw of 9.61 × 10^5^ Da showed regulatory effects on lipid metabolism and intestinal microbiota in T2DM (Yang et al., 2025). The red ginseng polysaccharide (RGP1-1) with a Mw of 5655 Da has a protective effect on the heart (Lian et al., 2022). Due to the large Mw and complex structure of red ginseng polysaccharides, studying the structure-activity relationship poses certain challenges. The conformation of polysaccharide molecules is generally believed to be related to their biological activity. Most known triple helix polysaccharides are *β*-glucans, which are characterized by the intermittent distribution of *β*-(1 → 6)-D-Glc branches on the main chain of the *β*-(1→ 3)-D-Glc pyran ring. However, the polysaccharide RGP1-1 isolated and purified from red ginseng is an uncommon triple helix pattern. The main chain of RGP1-1 is composed of →1)-*α*-D-Glc*p*-(4→ and →1)-*β*-D-Gal*p*-(4→ alternating connections and branches at the O-4 position to connect the *α*-D-Glc*p*-(1 → residue. The study did not delve into the relationship between this conformation and its ameliorative effect on oxidative stress injury in myocardial ischemia. Future research can introduce conformational perturbation experiments to clarify the main structure of red ginseng polysaccharides exerting cardioprotective effects, which will provide a basis for conformational based cardiovascular drug design.

Research has shown that the red ginseng polysaccharide RGP-AP-I is an acidic polysaccharide comprises a rhamnogalacturonan I (RG-I) backbone with repeating disaccharide units [ →2)-Rha*p*-(1 → 4)-GalA*p*-(1→] and three side chains substituted at the C(O)4 position of the rhamnose residue in the backbone. It exhibits enhanced immunostimulatory activity and has been shown to activate peritoneal macrophages, promote IL-6, IL-12, and TNF-*α* production (Lee et al., 2020; Lee et al., 2021). But the original study only conducted structural identification and activity verification, without degradation or modification controls, so there is no direct structure-activity relationship data itself. However, the immunomodulatory effects of some other plant polysaccharides can provide reference for the analysis of the structure-activity relationship of red ginseng polysaccharides. Based on the study of ginseng RG-I type pectin WGPA-2-RG, partial acid hydrolysis confirmed that its type II arabinogalactan (AG-II) side chain mainly mediates NO secretion and lymphocyte proliferation. Especially, the Ara residues attached to the molecular surface play an important role in enhancing lymphocyte proliferation and NO production by peritoneal macrophages (Zhang et al., 2012). Unlike ginseng WGPA-2-RG, which is mainly composed of AG-II side chains, RGP-AP-I exhibits a more complex side chain structure. The higher branching degree of *α*-1,5-arabinose and *β*-3,6-galactose in RGP-AP-I compared to WGPA-2-RG may increase the exposure density of terminal Ara residues, thereby enhancing the binding ability to macrophage pattern recognition receptors. However, it should be noted that excessive branching may lead to epitope shielding. Future research can verify the structure-activity of red ginseng RG-I after enzymatic digestion/chemical degradation to clarify the specific activity contribution of high branched Ara.

Existing literature suggests that monosaccharide composition, Mw, and glycosidic bonds may be important structural parameters affecting the biological activity of red ginseng polysaccharides. However, most of these associations are based on indirect evidence, and their universality and causality still need to be established through targeted structural modification and systematic activity evaluation. To overcome this bottleneck, there is an urgent need to establish a progressive technical path of “experimental confirmation, data integration, mechanism prediction” for the study of the structure-activity relationship of red ginseng polysaccharides. In the short term, sequential enzymatic hydrolysis and partial degradation are still reliable means to reveal the function of structural domains. In the medium term, a polysaccharide activity prediction model based on graph neural networks is expected to integrate fragmented literature data and establish universal rules for cross species structure-activity relationships. In the long run, with the improvement of sugar informatics databases and the maturity of enhanced sampling molecular dynamics methods, predicting biological activity from atomic structure will become possible. For red ginseng polysaccharides, the most urgent task at present is to establish a standardized structural descriptor system and biological activity dataset, laying the foundation for the implementation of the above methods.

## 9 Conclusion and prospect

Red ginseng, as a processed product of traditional Chinese medicine ginseng, contains a large amount of metabolites. At present, research on red ginseng is not only focused on saponin, but also on polysaccharides. Polysaccharides are large molecules with rich and diverse structures. Compared with other biological polymers such as proteins and nucleic acids, they have stronger ability to carry biological information, greater potential for structural variation, and higher safety. These characteristics make them important resources for human health and industrial innovation (Yang et al., 2025). Therefore, conducting research on polysaccharides from red ginseng has significant scientific, practical, and social value. Through reviewing existing literature, it is found that research on red ginseng polysaccharides is mainly focused on Korea and China, and their extraction and purification methods are mainly based on traditional water extraction and alcohol precipitation methods. Red ginseng polysaccharides are mostly acidic polysaccharides, which have immunomodulatory effects, anti-diabetic effects, anti-Alzheimer’s disease effects, health benefits for the skin, anticancer effects, and cardioprotective effects. These extensive pharmacological studies not only validate the traditional medicinal value of red ginseng but also explore and expand its new application directions. In summary, current research on polysaccharides from red ginseng mainly covers three aspects: extraction and purification, chemical structure, and biological activity. These studies have played a beneficial role in further developing red ginseng.

Although some progress has been made in the research of red ginseng polysaccharides, there are still many shortcomings in existing studies that need to be addressed in future research. At present, there are many methods for extracting and purifying plant polysaccharides, such as pulsed electric field extraction, deep eutectic solvent (DES), aqueous two-phase system (ATPS), three-phase partitioning (TPP), high-speed counter current chromatography, etc. (Yang et al., 2020; Zhang et al., 2023). The above technologies can be applied independently or form a combination strategy through complementary advantages. However, most laboratories still rely on traditional water extraction and alcohol precipitation methods to extract polysaccharides from red ginseng. Although the process is mature, this method has limitations such as long-time consumption, high energy consumption, and high temperature that may lead to polysaccharide degradation. A large amount of research is dedicated to exploring and optimizing innovative extraction techniques for phytochemicals (Wang et al., 2024; Lefebvre et al., 2021). In recent years, some new technologies have shown significant advantages in Panax plants, which can provide reference for the extraction of red ginseng polysaccharides. For example, DES combined with ATPS have been used for simultaneous extraction of polysaccharides and ginsenosides from *Panax quinquefolius* L. This method has high extraction efficiency, and the solvent can be recycled, while also being environmentally friendly (Zhou et al., 2022). Wu et al. (2025) successfully extracted *Panax quinquefolius* L. polysaccharides (AGP-DES-4) using the ultrasound-assisted deep eutectic solvents-based three-phase partitioning (UA-TPP-DES). And it has been proven that the solvent can be recycled at least 5 times while maintaining efficiency (Wu et al., 2025). However, the research on extracting polysaccharides from red ginseng using the aforementioned new technologies is still relatively scarce. Considering that *Panax quinquefolius* L. belong to the same genus and have certain similarities, these technologies can be introduced into the field of red ginseng polysaccharide extraction and purification in the future. Through targeted optimization of process parameters, a green preparation system for red ginseng polysaccharides that balances extraction efficiency, structural integrity, and environmental friendliness can be established. The study of the structure of compounds is a prerequisite for understanding their properties, functions, and potential applications. Due to the relatively late start of research on red ginseng polysaccharides and their complex structure, as well as the difficulty of separation and purification, current research on polysaccharides still lags small molecule compounds. The existing research mainly focuses on determining the molecular weight, monosaccharide composition, and glycosidic bond types of polysaccharides, and is still in the early stage of polysaccharide research. The study of advanced structures is relatively weak, and more promising technological means are needed to accurately analyze their advanced structures. The study of the structure-activity relationship of polysaccharides has always been a hot topic. Existing research uses artificial neural network (ANN) models for prediction and employs gradient weighted class activation mapping (Grad CAM) algorithm to explain the structure-activity relationship of raspberry polysaccharides (Lu et al., 2024). The study of the structure-activity relationship of red ginseng polysaccharides can draw on the ideas provided by existing research to provide theoretical basis for precise application.

The biological activity mechanism of red ginseng polysaccharides is complex, involving multiple pathways and targets. Although some studies have revealed some mechanisms of action, there are still many unknown areas that need to be further explored, especially the protective effect of red ginseng polysaccharides on the heart. Heart related diseases have become a major global health issue, and it is necessary to conduct more research on them, especially to find drugs for prevention or improvement from natural plants (Wal et al., 2024). Subsequent research requires the use of interdisciplinary approaches such as modern molecular biology and cell biology to systematically study the targets and signaling pathways of red ginseng polysaccharides at the cellular and molecular levels, improve the mechanism of their cardioprotective effects, and provide scientific support for the development of more targeted products. In addition, research on the synergistic effects of red ginseng polysaccharides with other polysaccharides can also be strengthened. According to existing research reports, the synergistic effect between polysaccharides can achieve complementary advantages, which is more effective than the application of a single polysaccharide, and even produces new functions that a single polysaccharide does not possess, significantly expanding the functions of polysaccharides. This can lay the foundation for the high-quality utilization of red ginseng resources.
